# Interesting Clinical Cases

**Published:** 1891-07

**Authors:** Wm. H. Doughty

**Affiliations:** Augusta, Ga.


### Southern Medical Record.

A MONHLY JOURNAL OF MEDICINE AND SURGERY.

VOL. XXI. ATLANTA, GA., JULY, 1891. NO. 7.

### Original Articles.

### INTERESTING CLINICAL CASES.

BY WM. H. DOUGHTY, M. D., AUGUSTA, GA.

Read before the Medical Association of Georgia, Forty-second Annual Session, Augusta, Georgia, April, 1891.

CASE 1.—*A Case of Arrested Pulmonary Tuberculosis* (?)

Well authenticated cases of recovery from pulmonary tuberculosis by arrest of the infection are so rare that practically its mere mention is calculated to excite incredulity. It is in deference to this, which involves the suspicion of a possible error of diagnosis, that I have placed an interrogation mark as a suffix to the title of the case reported. The diagnostic test is the demonstration with the microscope of the presence of the bacillus tuberculosis, now known and recognized as the uniform cause of phthisis pulmonalis; Nevertheless, it is not always avaiable, especially in the very early stages, when cough is slight and with little or no expectoration. Clinically in this class of cases we are still left dependent upon signs and symptoms for a reliable diagnosis; the microscopic examination of the sputum confirming or rejecting it at a later period. At present Koch's lymph (parataloid) is used for diagnostic purposes also; its established reaction in tubercular subjects becoming, however, only an addition to other clinical observations, although deemed somewhat specific. The curability of tubercular phthisis as a clinical problem, like that of cancer, involves the further question of a certainty of the diagnosis which, in its initial stages, is with difficulty m ıd ·. Hence, the doubt in all cases, like the one now reported. Therapeutic measures for its arrest inspire little confidence, although improvement in the condition of the patient, with consequent retardation of the disease in its progress, is not unfrequently obtained by their judicious use for the relief of attendant catarrhal and inflammatory conditions. Rationally a recovery, in the sense of a cure, depends upon the destruction of the bacillus, or its circumscription in the tissues so as to render it inocuous and non-reproductive. What was the fate of the bacillus in the present case, if it really existed, cannot be stated, but nearly three years have elapsed since the case was under observation, the patient remaining in good health.

Stro npell, in his invaluable "Text Book of Medicine," says: "Apex-catarrhs are as a rule tubercular." Allowing six or eight months for the feeble bacillus to inaugurate the disturbances due to its lodgment and multiplication in the apex of the lung, in a subject apparently free from hereditary predisposition, the conviction forces itself upon me, that the infection existed, and, moreover, that its arrest was due to the treatment, as will further appear.

Miss———, aged 17, died on May 12th, 1887, from phthisis, after an illness of eighteen months or more. She was a girl of feeble constitutional habit; tall, rapid in her growth; of sedentary habits; capricious in her tastes; always a delicate eater; one hard to feed at best. The disease began in the apex of the right lung as a limited catarrh, and throughout was characterized by a slight cough (dry), and with scanty expectoration—no hemorrhages, but little pain. Even in the latter months these peculiarities obtained, notwithstanding the physical examination of the chest indicated a cavity in the apex of the right lung, with well marked bronchovesicular respiration throughout both lungs. There was a remarkable absence of moist rales, with scanty expectoration, frequent pulse, abnormal temperature, general cachexia, with progressive emaciation, slight diarrhœa, and corresponding general debility.

Her mother was her constant attendant and nurse (she being an only child), and throughout her illness maintained the most intimate personal relations to her, both day and night. A woman of large frame, 42 years of age, and hitherto of very active habits of life, her devotion to her afflicted daughter was not only constant, but injurious. However, after the death of her daughter, she led a more active life again. Returning from a trip north in the fall, she appeared to be in good health, having recovered from the fatigue produced by long nursing. She continued so until about 1st January, 1888, when slight nausea and the suppression of her courses excited the apprehension of pregnancy. She had been regular and well since the birth of her late unhappy daughter, never having conceived since. At her time of life and after a vacation (honest) of sixteen or more years, she was filled with apprehension for her safety. Time confirmed the presumption of pregnancy, and she was delivered of a seven-month's child on June 28th, 1888, after an easy, quick labor, precipitated by no obvious cause. The child was born in a state of suspended animation, from which it was with difficulty resuscitated; lived only ten days, a feeble, unpromising, premature infant, conceived in grief and nurtured by a tuberculous mother, as will now appear.

During gestation all went well with the mother until about 15th February, when I was asked to see her. I found her having slight, dumb chills, recurring about mid-day. There was no cough, sore throat, or anything pointing directly to pulmonary trouble. She was apparently suffering from malarial chills (she lived in a malarious locality), for which quinine was freely given. Arrested for a time, they recurred weekly, and with these recurrences there came, ultimately, first, a slight, dry cough, with sore throat; a frequent irritable pulse; slight permanent elevation of temperature; night sweats, and manifest loss of flesh, that provoked in her mind the fixed apprehension that she, too, was developing consumption. There was little to confirm this apprehension in the family history, particularly with one of her age (42), but it soon became apparent that a differential diagnosis between acute tuberculosis and recurring malarial fever (intermittent) must be made. The failure of *quinine*, freely used, to control the fever, together with increasing dry cough, frequent pulse, with slight, constant elevation of temperature, etc., supplemented by the following physical signs, established the existence of the former.

APEX OF RIGHT LUNG.—Broncho-vesicular respiration; high-pitched, prolonged expiration; slight dullness on procussion; appreciable bronchophony, especially behind, above the supra-spinous ridge. Balance of right lung, with the entire left, being apparently normal.

She was informed that phthisis was in course of development, as in the case of, and probably contracted from her daughter. The following treatment was advised: Nutritious food in abundance, open air exposure, and 5 m. of Fowler's solution, with 5 gr. capsule of quinine three times daily, after eating, regularly and *indefinitely*. This treatment was pursued religiously for five months, or until about August, when it was discontinued, the patient being to all appearances well, and, in the meantime, having had a premature labor, as before stated. After its employment for a few weeks, her fever disappeared, cough abated and ultimately ceased, appetite and spirits improved; she gained flesh and strength, and her complexion regained the hue of health, temperature became normal, and the "catarrh" disappeared. In short, she seemed a well woman, pregnancy excepted. Early in August, she went north in good health.

Now the question arises, was there a tubercle-inoculation in this case with an arrest by treatment?

The contagiousness of phthisis under appropriate circumstances is no longer a question sub-judice; the literature of the subject abounds in instances so conclusive that there is no room for doubt, particularly since the tubercle-bacillus has been identified as the contagium vivum. The case reported, in its individual character, especially the exposure of the subject to contagion and the definite localization of the disease in a limited portion of the lung, will bear comparison to an attempt at a tubercle culture in the human subject, successful up to a certain point and then arrested, possibly by the destruction of the bacillus, or by some change in the culture medium unfavorable to its further development or existence. I entertain no doubt of the disease having been communicated to the mother from or by the daughter, but the fact of its arrest (if it be one), is what has induced its presentation. I cannot escape the conclusion that is was arrested, cured if you please, by the treatment, and this alone throws doubt upon the diagnosis. In other words, its arrest becomes an argument against a true, genuine infection or inoculation by the tubercle-bacillus.

---

CASE 2.—*Obstruction of Both Parotid Ducts, with Consequent Dilatation of the Same, and Enlargement of the Parotids.*

In July, 1889, I was consulted by Miss A—— about a painful swelling under each ear, greater on the left side than on the right; painful to touch and with every movement of the jaw, and at times accompanied with a circumscribed redness on the left cheek that lasted for several days. This condition had existed for a long time, and was gradually increasing. Patient remarked that on pressing the swelling on either side it caused an immediate flow of saliva beside the plate of false teeth in use by her.

Upon examination I found that she was wearing a full set of upper teeth, and, as stated, when pressure was made upon the enlarged glands, an escape of the salivary secretion took place. Removing the plate and renewing the pressure there was a visible jet or stream of the secretion produced as often as the pressure was relaxed and renewed. Closer examination revealed the fact that the mucous membrane, at and around the orifices of the ducts, was in a state of chronic inflammation, caused by the artificial plate, which seemed to be too wide, and thus produced a partial stenosis of the ducts at this point, which only under pressure permitted the flow of the secretion. There followed an enormous dilatation of the ducts, with injurious pressure upon and enlargement of the parotids. Steno's duct is 2 1-2 inches long, dense in structure, and about the size of a " crow quill, " and is, perhaps, capable of a degree of dilatation beyond what would ordinarily be calculated. It must have been very great in this case, as indicated by the size of the stream of fluid discharged under pressure, and the frequency with which it could be produced in large quantity· Catheterization of the duct is a practicable measure. The interest of the case centres in its bi-lateral character, and in its cause, the wearing of an imperfectly fitting plate of false teeth. although the subject made no complaint of discomfort from it. She was referred to our accomplished dentist, Dr. George A. Patrick, for the correction of the defect in the adjustment of the plate, and for further treatment.

---

CASE 3.—*Case of Internal, or Pelvic Spina-Bifida.*

I present this case merely to record it in the transactions of this Association as one of phenomenal interest. Its full history will doubtless be given to the profession by Prof. T. Gaillard Thomas, the distinguished gynæcologist of New York, to whom it was referred for a differential diagnosis from other forms of pelvic tumor. His exact diagnosis of "Pelvic Spina-Bifida" I have since confirmed, by a careful examination. As far as my investigations go, it is the fourth in a series of published cases—one by Dr. Emmett, in 1870, and two others by Thomas, in 1885. This is sufficient to indicate the rarity of the lesion, and will doubtless prove specially interesting to the gynæcologists of this Association, who, like myself, may at any moment be called upon, unexpectedly, to differentiate it from other tumors. For their instruction, I will quote the concluding sentences of a summary of these cases, (two of which terminated fatally under operative interference, performed without a clear comprehension of their exact nature,) published by Thomas, in Gilliard's Medical Journal, March, 1885.

"Although our knowledge concerning this form of pelvic tumor is at present so meagre, we may, I think, even now deduce the following lessons from the two fatal cases recorded in this essay, which will prove useful for the prevention of a very possible increase in the number of similar unfortunate ones."

1. "When cyst is found in the pelvis, behind the rectum, filling the hollow of the sacrum, apparently attached to that bone, let the diagnostician carefully exclude the possibility of its being spina-bifida before interfering with it."

2. "If it be decided to interfere with such a tumor, let a small portion of fluid be first drawn by a hypodermic needle, and if this be found to be a limpid, non-albuminous fluid, let the probabilities of the sac being connected with the meninges of the cord receive due consideration, and guard against further interference."

